# Supplementary material for: Intelligent image-based in situ single-cell isolation
Source: Nat Commun. 2018 Jan 15;9:226. doi: 10.1038/s41467-017-02628-4 (PMC5768687; doi:10.1038/s41467-017-02628-4)
Supplement: Supplementary file 1 — Supplementary Information [file 41467_2017_2628_MOESM1_ESM.pdf]

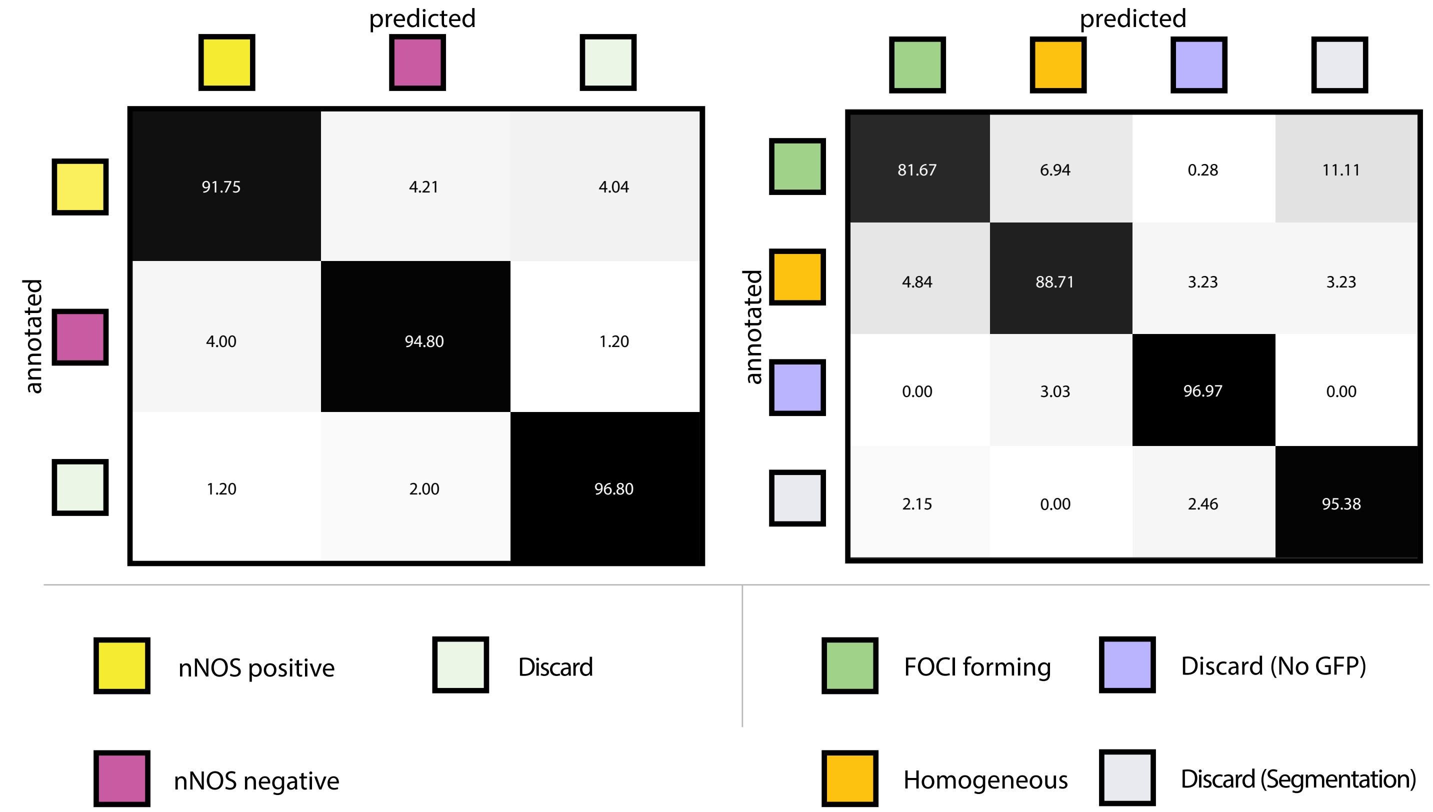

10-fold cross-validation and confusion matrices for a random forest classifier classifying nNOS+/nNOS-/trash (left, n=157) and foci-forming/homogeneous/no-gfp/trash for the last experiment (right, n=165).

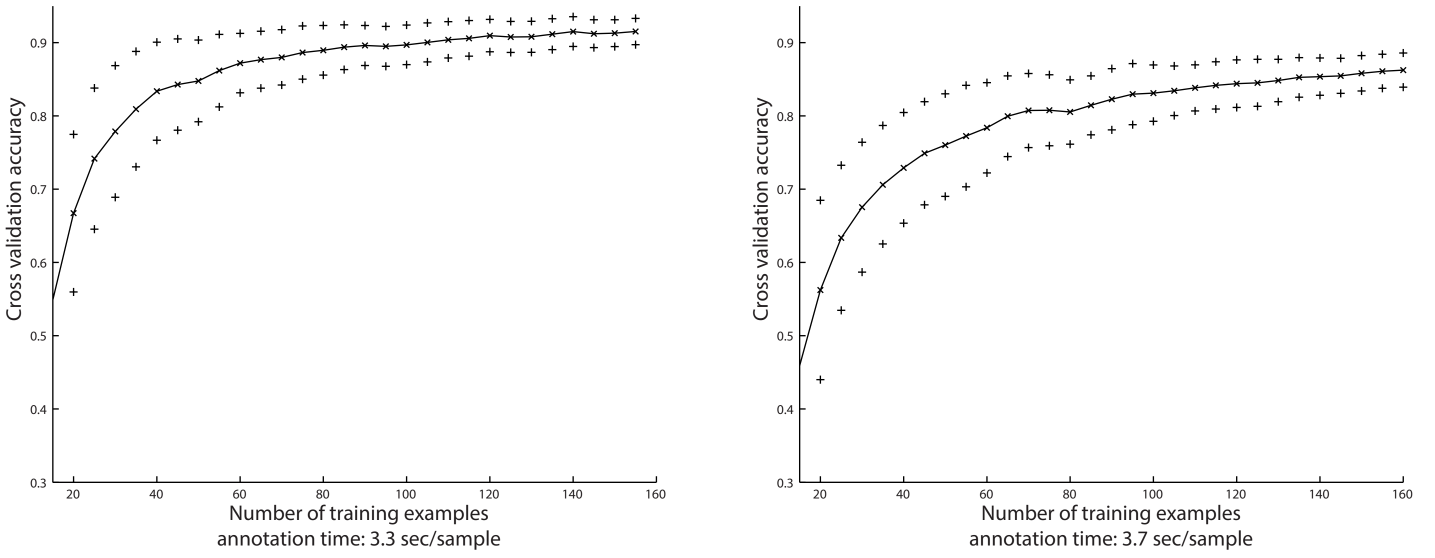

A randomly selected set of annotated samples were used for cross validation. Error bars depict the mean and standard deviation of cross validation accuracies (n=100).

Supplementary Figure 1 - quantitative evaluation of machine learning performance

Image ID: 116

Image

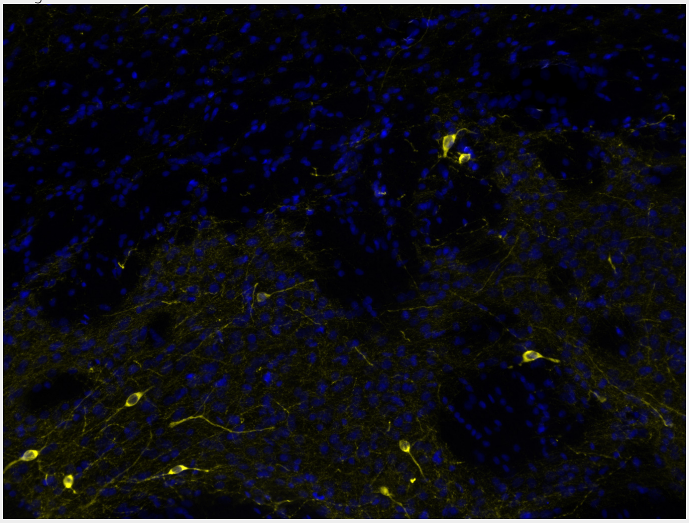

Image ID: 116
Screen ID: **csilla_nm_20161021_002**
File name: nnos-20161021-002_wA01_f29_z0_t0_c0.png
Position (m): [0.0128325,0.00610237]

Go back

CAMI Online
Computer Assisted Microscopy Isolation

Index page   Experiments   Screens

nnos-20161021-002-nNOS-positives-20161103

Experiment

Experiment ID: 19
Name: nnos-20161021-002-nNOS-positives-20161103

Description: nNOS cells from experiment nnos-20161021-002

Update

Screen: **csilla_nm_20161021_002**
Uploaded: 2016-11-03 17:16:04.0
Last cut:
Last edited:

Cells

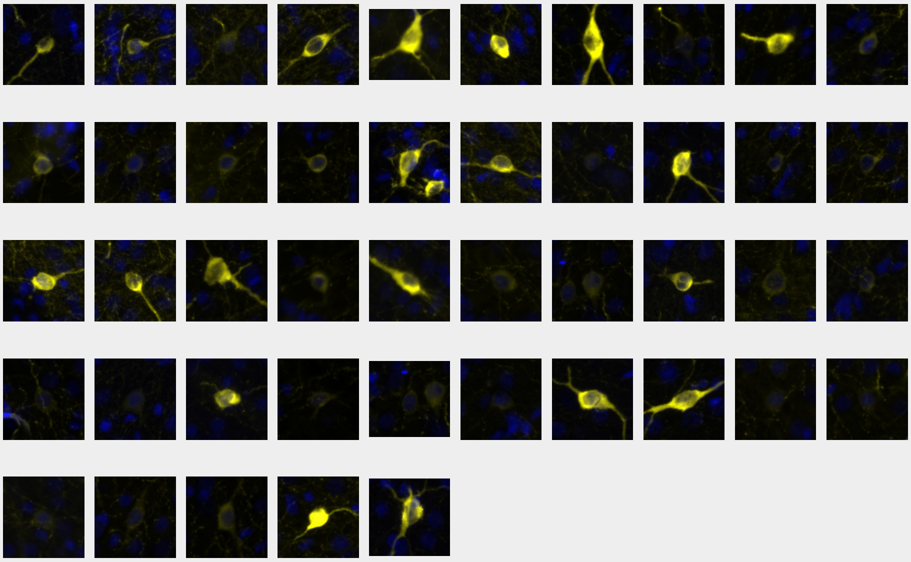

Generate contours of selected cells   Select all   Deselect all

Go back

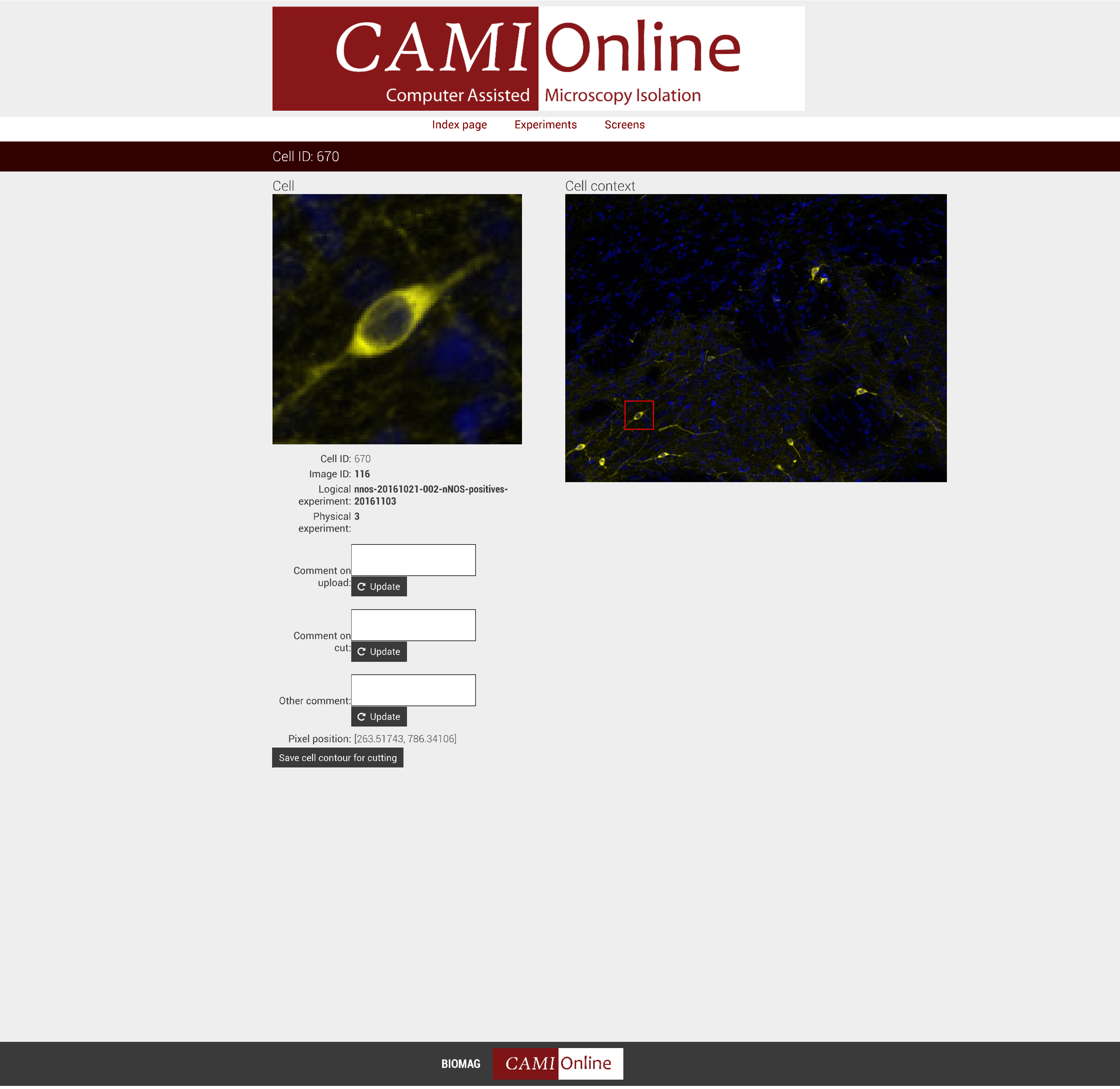

Supplementary Figure 2 - CAMI online database

Supplementary Note 1 – Laser-etched landmarks and registration evaluation

As a first step for every experiment, we etched 50x50 µm landmarks into poly-L-lysine coated slides (1 landmark per slide) using a microdissection microscope (Zeiss Axio Observer microscope with PALM MicroBeam manipulator). The landmarks fix positions that allow us to register image data between microscopes. They were designed to indicate the orientation to avoid any errors due to rotation of the coordinates. Unique barcodes may also be etched into the slide to identify samples.

The landmarks are first etched into the slides in a sample preparation step. In the high content microscope images, the landmarks are used to register the coordinates of the detected cells and contours selected for isolation.

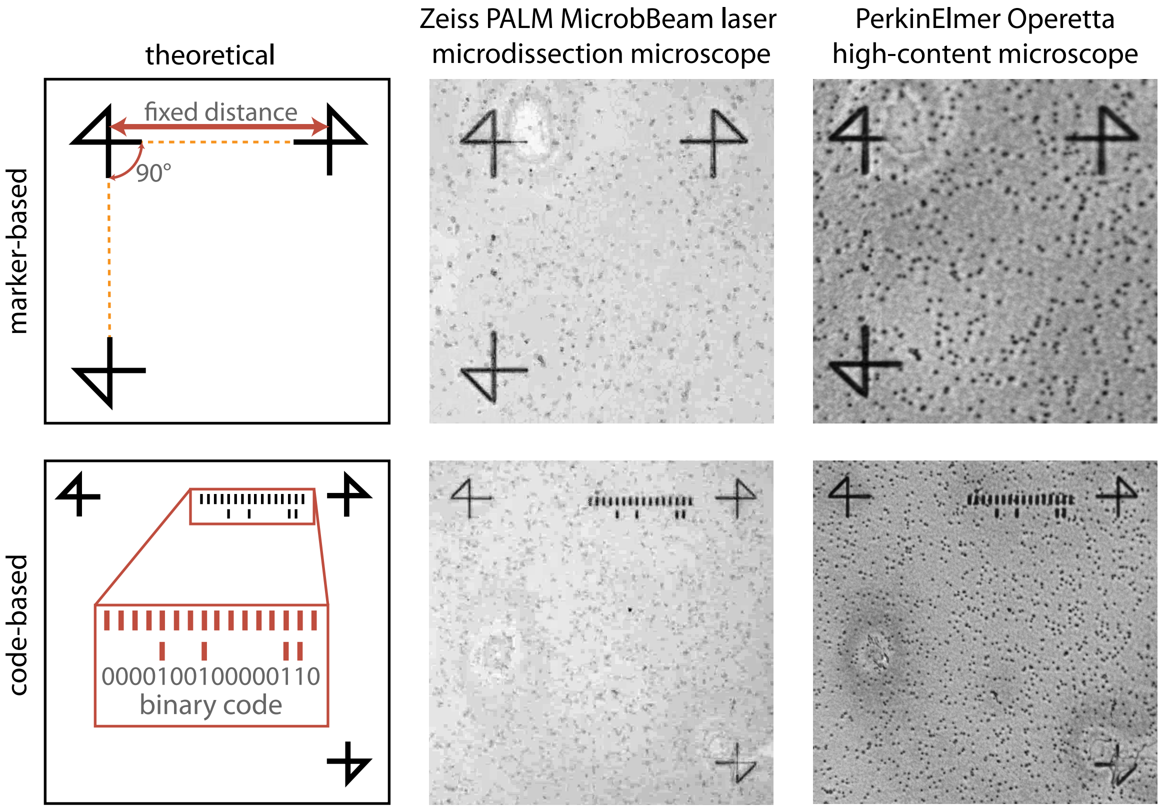

Supplementary Figure 3. Calibration landmarks.

Evaluation of registration error

Registration of the coordinate systems between the laser microdissection microscope and the high content microscope is imperfect. There are two main sources of error: 1) small scale differences between coordinate systems, 2) rotation, because it is impossible to consistently align the slide in different microscopes.

We evaluate the accuracy of the landmark registration by etching two grid patterns, a fine pattern and coarse pattern, into a glass slide using the Zeiss PALM microscope with 63x objective. The grids were manually located in the second microscope and those locations were compared to the theoretic locations determined by using the landmarks.

The grid widths were 20 µm and 200 µm respectively. The aim was to simulate typical working conditions with the fine pattern (in which cells are densely crowded) and the coarse pattern aims to simulate larger areas of interest.

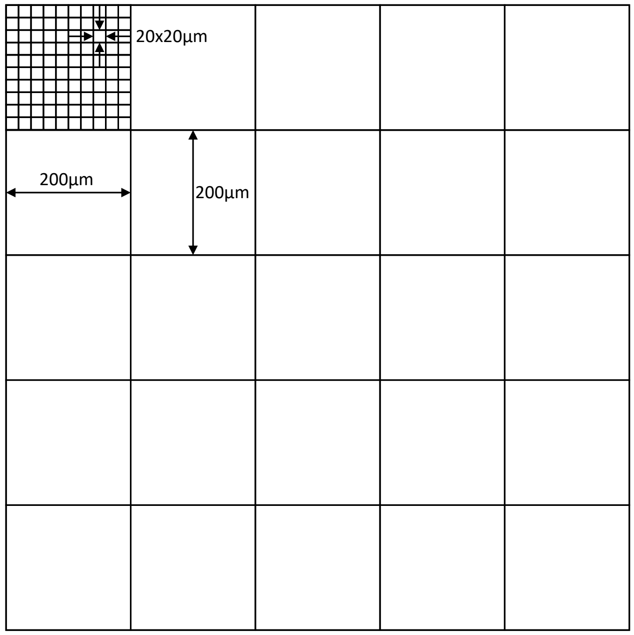

Supplementary Figure 4. Fine and course grid calibration patterns.

High and low magnification images of the patterns acquired by using the ZEISS PALM microscope are shown in Supplementary Figure 5.

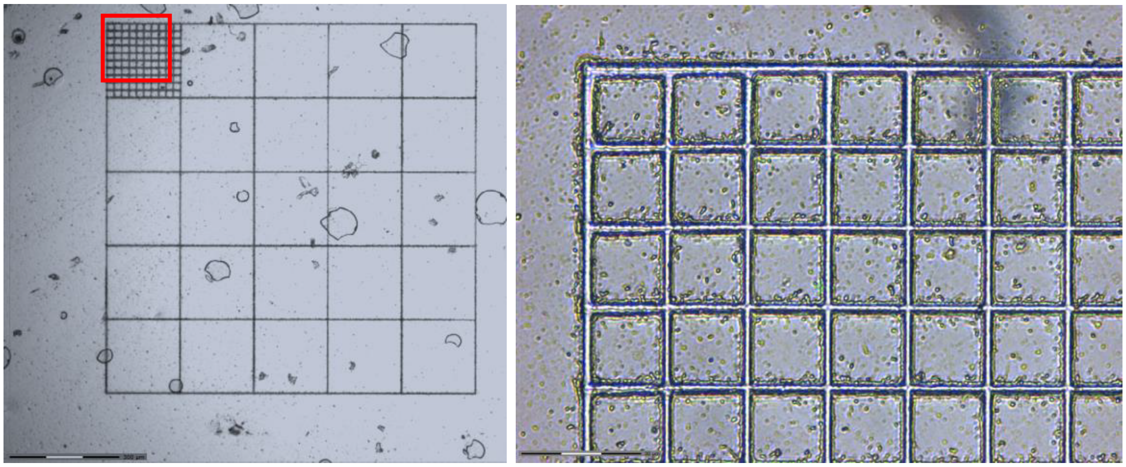

Supplementary Figure 5. Grid patterns imaged with Zeiss PALM using 5x (left) and 63x (right) magnification. Scalebars 300 µm and 30 µm respectively.

The sample was then imaged using the PerkinElmer Operetta microscope, with 60x long working distance objective. A montage of the images is shown in Supplementary Figure 6.

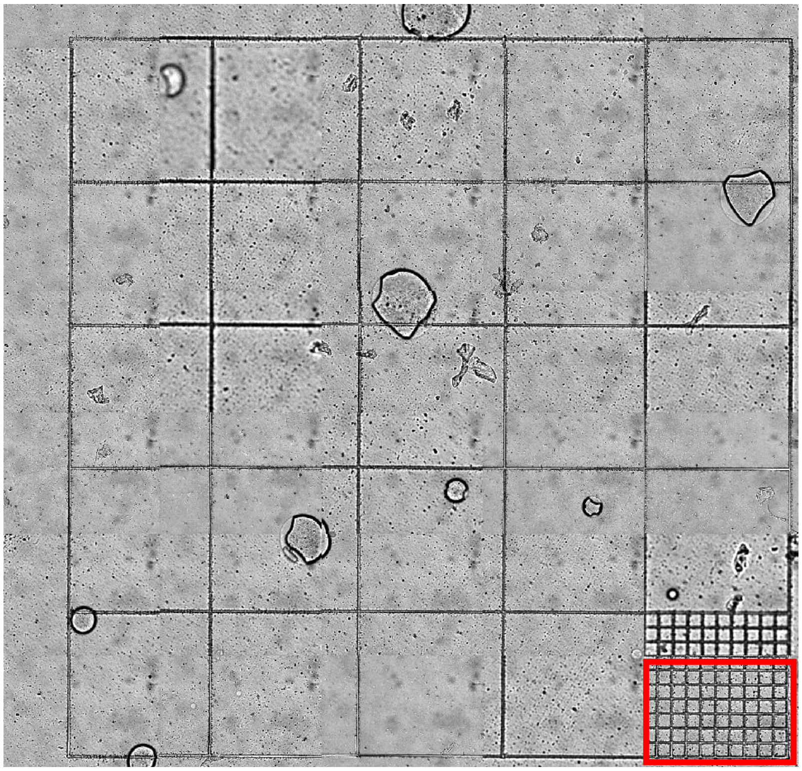

Supplementary Figure 6. Montage of the grid pattern measured on the PerkinElmer Operetta microscope using 60X magnification.

We manually located all the corners of the grid patterns using the PerkinElmer Operetta microscope by zooming and panning. The measurements were made to pixel precision. A snapshot is shown in Supplementary Figure 7.

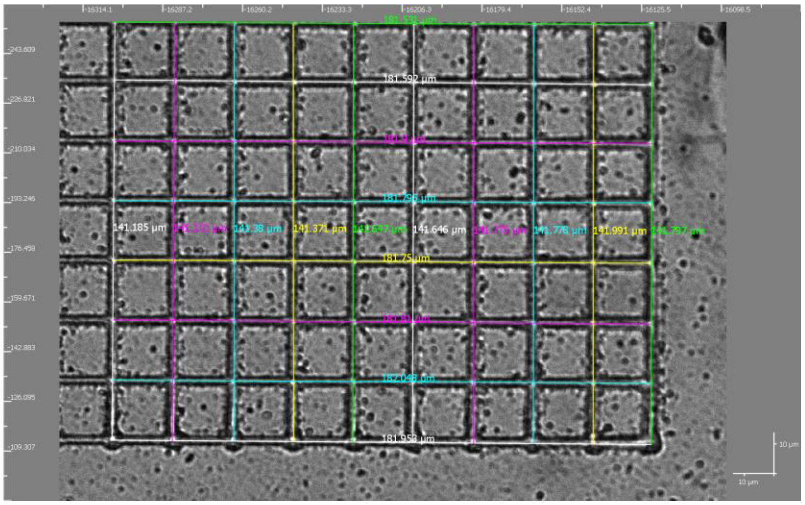

Supplementary Figure 7. Manual location of the fine grid pattern marked in Supplementary Figure 6.

The manually located grid corners were then compared to the theoretic locations determined by using one of the corners as a landmark. The registration errors are shown as vector fields in Supplementary Figure 8. The mean registration error for the fine grid pattern was 1.7 µm and the mean error for the coarse grid pattern was 4.3 µm.

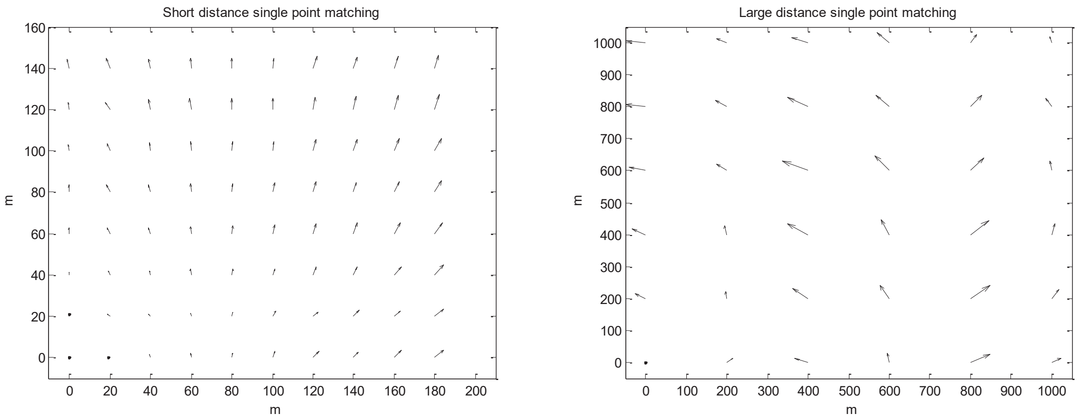

Supplementary Figure 8. Error vector flows. Fine grid pattern errors (left) and coarse grid pattern errors (right). For visualization, error bars are scaled up by a factor of 10.

More accurate registration can be achieved using more landmarks. With two landmarks, it is possible to accurately estimate translation and scale, and with three landmarks it is possible to accurately estimate translation, scale, and rotation. For our experiments, accuracy was deemed sufficient with a single landmark. In Supplementary Figure 9, we show the registration error fields for two and three landmarks.

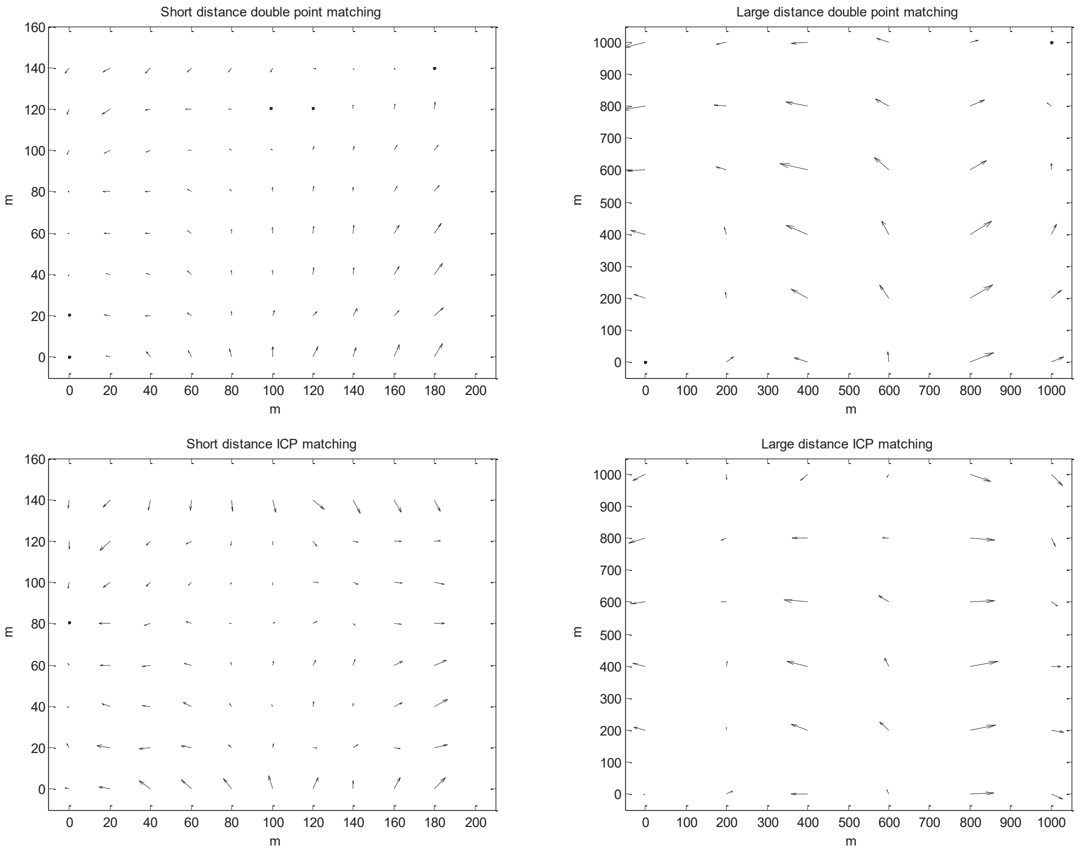

Supplementary Figure 9. Error vector flows for registration with 2 landmarks (top row) and 3 landmarks (bottom row). Fine grid pattern errors appear on the left and coarse grid pattern errors appear on the right. For visualization, error bars are scaled up by a factor of 10.

Finally, we show the computed mean registration error using 1, 2, and 3 landmarks for both the fine and coarse grid patterns in Supplementary Figure 10.

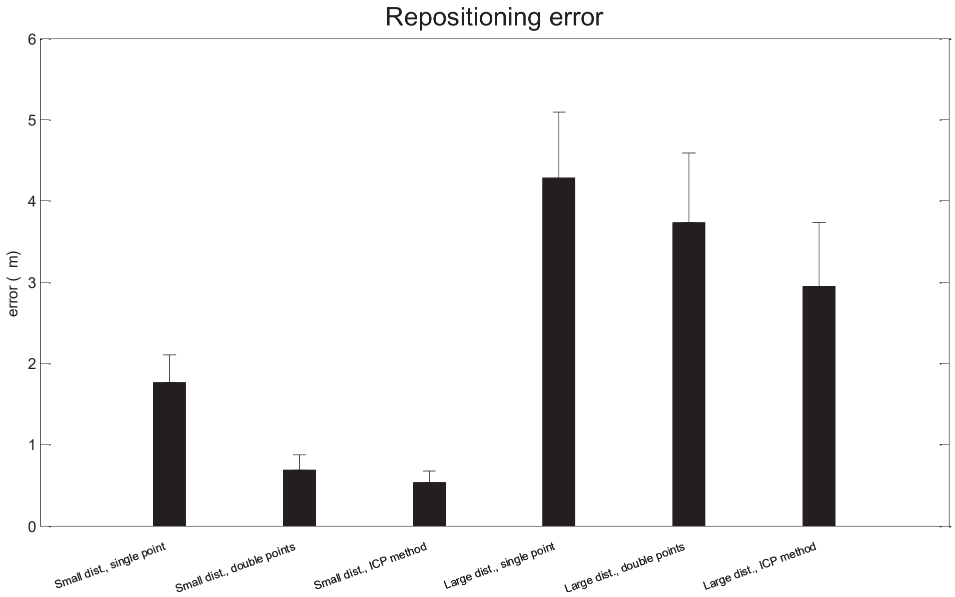

Supplementary Figure 10. Mean error after registration using 1, 2 , or 3 marker points on fine and coarse grid patterns.

In conclusion, if the working area is near the landmark, a single landmark has sufficiently accurate registration. Two or three landmark registration significantly reduces registration error for applications where it is necessary.

Supplementary Note 2 - Multi-layer 'gas of near circles' image segmentation model*

Image formation model for fluorescent microscopy. The image formation model we assume is $I_{\text{observed}} = I_{\text{background}} + I_{\text{original}} * V$, where V might be some illumination problem, which is corrected using the CIDRE method[2]. $I_{\text{background}}$ is a nearly flat non-zero surface with negligible noise (so called dark noise). We assume that the measured intensity is proportional to the number of fluorescent particles and therefore, using low numerical aperture objectives, cells on top of each other result approximately multiple intensity values. We denote the intensity of a single cell by $\Delta\mu = \mu_+ - \mu_-$, and its variance by $\Delta\sigma^2 = \sigma_+^2 - \sigma_-^2$, where μ_+, σ_+^2 and μ_-, σ_-^2 are the mean and variance of a single object and the background respectively. Multiple cells produce $\mu_- + n\Delta\mu$ intensity and $\sigma_-^2 + n\Delta\sigma^2$ variance. Intensity parameters are estimated on corrected images using maximum likelihood estimation.

Multi-layer phase field model of a 'gas of near-circles'. A phase field function ϕ is a level set representation: given a threshold z it determines a region R in the image domain $\mathcal{D}$: $R = \{x \in \mathcal{D} : \phi(x) \geq z\}$.

The nonlocal phase field energy used to model a gas of non-touching, non-overlapping near-

*This model was originally published in [1]

circular objects has the following form:

$$E_f(\phi) = \int_{\mathcal{D}} \left\{ \frac{D_f}{2} |\nabla\phi|^2 + \alpha_f \Big(\phi - \frac{\phi^3}{3}\Big) + \lambda_f \Big(\frac{\phi^4}{4} - \frac{\phi^2}{2}\Big) \right\} - \frac{\beta_f}{2} \iint_{\mathcal{D}\times\mathcal{D}'} \nabla\phi \cdot \nabla\phi' \, G(x - x') \ , \quad (1)$$

where (un)primed functions are evaluated at ($x \in \mathcal{D}$) $x' \in \mathcal{D}' \equiv \mathcal{D}$. The interaction function $G : \mathbb{R}^2 \to \mathbb{R}$ controls the range of interaction. The model can therefore be used in place of a classic active contour, but with the concomitant advantages of the phase field framework.

The above model is appropriate when object instances are well-separated, but it has a severe limitation: it cannot represent touching or overlapping object instances, because a phase field function represents subsets of $\mathcal{D}$; and the nonlocal term in the energy, as well as generating the desired near-circular shapes, also causes object instances with small separation to have high energy.

To remove these difficulties, a multi-layer version of the above model was developed in [3]. Let $\tilde{\phi} = \{\phi^{(i)}\}_{i\in[1..\ell]} : [1..\ell] \times \mathcal{D} \to \mathbb{R}$, where ℓ is the number of layers. The energy $\tilde{E}_f(\tilde{\phi})$ of the model is simply the sum of energies of independent layers extended with a term that penalizes overlapping pairs of object instances by an amount proportional to overlap area:

$$\tilde{E}_f(\tilde{\phi}) = \sum_{i=1}^{\ell} E(\phi^{(i)}) + \frac{\kappa}{4} \sum_{i\neq j} \int_{\mathcal{D}} (1 + \phi^{(i)})(1 + \phi^{(j)}) \ , \quad (2)$$

where κ controls the overlap penalty. This model solves the two issues mentioned above. Overlapping object instances can now be represented by appearing in different layers, and the inter-object repulsion is now removed because it is energetically favourable for nearby objects to be represented

on different layers, thus incurring no energy penalty.

Datam model for overlapping cells. We model the image intensities in the background and in the single-object foreground (*i.e.* a cell) as having fixed (but different) means and variances, leading to Gaussian distributions with independent pixel intensities by maximum entropy. When several objects overlap, we model the resulting image as the sum of the intensities from the background and each of the overlapping objects, so that the resulting model is again Gaussian with independent pixels, but with mean and variance equal to the sum of the means and variances of the background and the objects.

We define $\phi_+ = \sum_{i=1}^{\ell} \frac{(\phi^{(i)}+1)}{2}$, which represents the number of overlapping objects at each point ([1]). Then the likelihood energy is

$$E_{\text{intensity}}(I, \phi_+) = \int \frac{(I - (\mu_- + \Delta\mu\phi_+))^2}{2(\sigma_-^2 + \Delta\sigma^2\phi_+)} \ , \tag{3}$$

where I is image intensity; μ_- and σ_-^2 are the mean and variance of the background; and $\Delta\mu$ and $\Delta\sigma^2$ are the changes in mean and variance brought about by each new overlapping object.

Pre-processing Iit was shown [1] that the 'multi-layer gas of circles' model resutls more accurate segmentation by using initial seeds inside the object to be segmented. To find nuclei centers in "difficult images" an A-Trous wavelet transform based spot detection were used [4]. The computational complexity is proportional to the number of 'gas of circles' layers, we have to minimize the number of these layers. For that we chose a graph/map coloring method as follows. Let each seed centroid be a node in a graph. An edge connects two nodes if they are close (this distance was set

to 3.5 times the preferred object radius). After building the graph we can color the nodes with 4 color to provide the none of connected node pairs has the same node color.

Post-processing To avoid 'degenerate' configurations the following was applied:

- Discard embedded smaller objects. All objects that is completely surrounded by any other object on an other layer, is removed.
- Merge fully overlapping objects. If the Jaccard index of two objects from different layers is bigger than 0.80, they are merged.
- Discard objects toucing the borders.

CellProfiler modules The segmentation method extended with the pre- and postprocessing steps are implemented in Matlab and Python as modules compatible with CellProfiler v1.0 and v2.0 respectively.

- IdentifyPrimaryMLGOC
- IdentifySecondaryMLGOC
- MeasureObjectAreaShapeMLGOC
- MeasureObjectIntensityMLGOC
- MeasureTextureMLGOC

- ConvertToImageMLGOC
- SaveImagesMLGOC

Figures

1. C Molnar, IH Jermyn, Z Kato, V Rahkama, P stling, P Mikkonen, V Pietiinen, P Horvath, *Sci. Rep.* 6, Article number: 32412 (2016).
2. K Smith, Y Li, F Piccinini, G Csucs, Cs Balazs, A Bevilacqua, P Horvath, *Nat. Methods* 12, 404-406 (2015).
3. C Molnar, Z Kato, IH Jermyn, *ICPR*, 1427–1430, (2012).
4. Olivo-Marin, J.-C., *Pattern Recognit*, **35**(9), 1989–1996 (2002).

Supplementary Note 3: Timing analysis of the CAMI pipeline

Duration of all intermediate steps for cell isolation were measured and reported. Supplementary Table 1 provides a summary of the time cost for each step of cell isolation from one slide, organized into three phases: imaging, analysing, and isolation. Some steps such as the high content imaging setup have a fixed cost per slide (finding focus, locating the sample, etc.), while other steps depend on the number of cells isolated.

	Step	Time (sec)	per unit
Imaging	Marker burning	360	slide
	High content imaging setup	1200	slide
	High content imaging	1.2	image
Analysis	Data export	0.4	image
	Image segmentation and machine learning	6.9	image
	Contour export	240	slide
Isolation	Laser microdissection	10.4	cell
	Catapulting and checking	3	cell
	Stage alignment for next cell	0.2	cell

Supplementary Table 1.

The number of images and number of isolated cells vary between experiments, which affects the total time cost. Below, we provide four examples of isolation experiments with different numbers of isolated cells and acquired images. The time costs for these experiments are provided in Supplementary Table 2.

Example 1. Number of images: 150, number of isolated cells: 50
Example 2. Number of images: 1000, number of isolated cells: 50
Example 3. Number of images: 200, number of isolated cells: 500
Example 4. Number of images: 2000, number of isolated cells: 500

	**1**	**2**	**3**	**4**
**Number of images/cells**	150/50	1,000/50	200/500	2,000/500
**Imaging**	1,740	2,760	1,800	3,960
**Analysis**	1,335	7,540	1,700	14,840
**Isolation**	680	680	6,800	6,800
**Total (sec)**	3,755	10,980	10,300	25,600
**Total (h)**	1.04	3.05	2.86	7.11

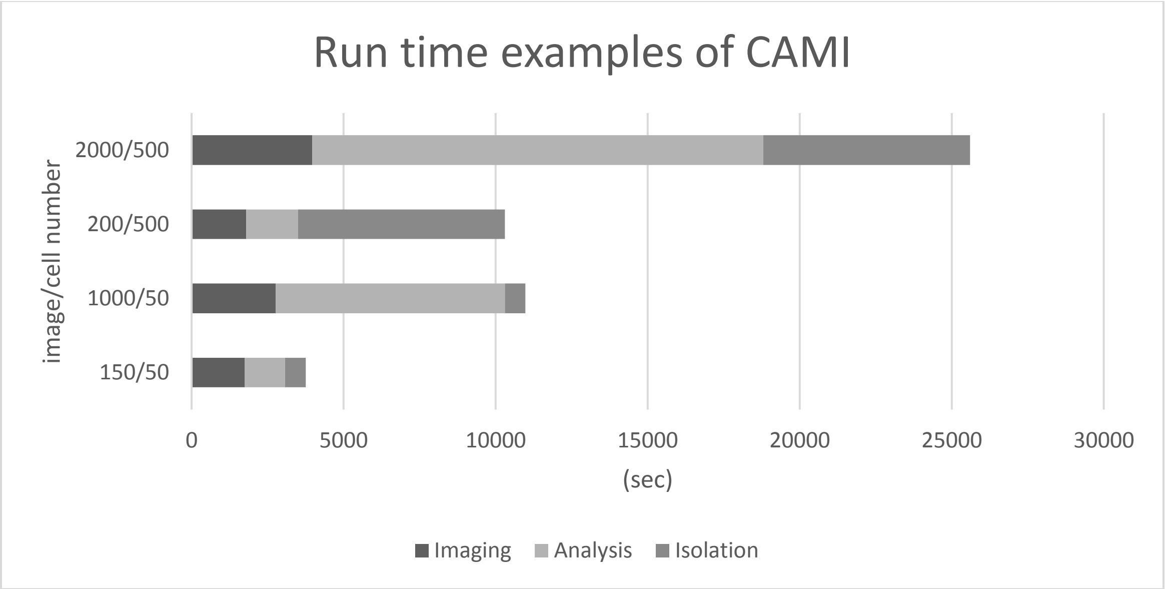

Supplementary Table 2.
