# Supplementary material for: Intelligent image-based in situ single-cell isolation
Source: Nat Commun. 2018 Jan 15;9:226. doi: 10.1038/s41467-017-02628-4 (PMC5768687; doi:10.1038/s41467-017-02628-4)
Supplement: Supplementary file 3 — Description of Additional Supplementary Files [file 41467_2017_2628_MOESM3_ESM.pdf]

Description of Additional Supplementary Files

File Name: Supplementary Data 1
Description: Single-cell digital PCR results comparison.

File Name: Supplementary Data 2
Description: Comparative results of independent whole-transcriptome analyses.

File Name: Supplementary Data 3
Description: Transcriptome statistics.

File Name: Supplementary Software
Description: CellProfiler implementation of the multi-layer gas of overlapping circles model. CellProfiler pipelines and modules for the presented experiments. Advanced Cell Classifier v2.1. CAMIO web portal installation files.
