# Supplementary material for: Intelligent image-based in situ single-cell isolation
Source: Nat Commun. 2018 Jan 15;9:226. doi: 10.1038/s41467-017-02628-4 (PMC5768687; doi:10.1038/s41467-017-02628-4)
Supplement: Supplementary file 7 — Supplementary Software [file 41467_2017_2628_MOESM7_ESM.zip › SuppSW3_ACC_v2_1.zip!/ACC_Documentation.docx]

**Advanced Cell Classifier (ACC) – user manual**

www.cellclassifier.org

**v2.1 - Sept 2016**

(written by Filippo Piccinini & Tamas Balassa)

**Prof. Peter Horvath, PhD**

Synthetic and Systems Biology Unit

Biological Research Center of the

Hungarian Academy of Sciences

horvath.peter@brc.mta.hu

**INDEX**

| 1 | **Brief description** | p. | 4 |
| --- | --- | --- | --- |
| 2 | **License** | p. | 4 |
| 3 | **System requirements** | p. | 5 |
| 4 | **Installation** | p. | 5 |
| 5 | **Input dataset structure** | p. | 6 |
|  | 5.1 Image nomenclature | . | . |
| 6 | **Getting started** | p. | 9 |
| 7 | **Graphical user interface** | p. | 11 |
|  | 7.1 Cell view | . | . |
|  | 7.2 Image selector | . | . |
| 8 | **File menu** | p. | 13 |
|  | 8.1 New, open, and save project | . | . |
|  | 8.2 Add and remove plates | . | . |
|  | 8.3 Import trained data and change settings | . | . |
| 9 | **Visualization** | p. | 15 |
| 10 | **Annotation** | p. | 17 |
|  | 10.1 Create, delete, load, and save classes | . | . |
|  | 10.2 Manual annotation | . | . |
|  | 10.3 Random annotation | . | . |
|  | 10.4 Find similar cells | . | . |
|  | 10.5 Active learning | . | . |
|  | 10.6 Phenotype finder | . | . |
|  | 10.7 Generate sampling pool | . | . |
| 11 | **Classification** | p. | 26 |
|  | 11.1 Classification settings | . | . |
| 12 | **Output** | p. | 27 |
|  | 12.1 Predict current image | . | . |
|  | 12.2 Predict selected plates | . | . |
|  | 12.3 Feature-based statistics | . | . |
| 13 | **CL2M – Cell uploading module** | p. | 35 |
| 14 | **ACC and CellProfiler** | p. | 35 |
|  |  |  |  |
|  |  |  |  |
|  |  |  |  |
|  |  |  |  |
|  |  |  |  |
|  |  |  |  |
|  |  |  |  |
|  |  |  |  |
|  |  |  |  |
|  |  |  |  |
|  |  |  |  |
|  |  |  |  |
|  |  |  |  |
|  |  |  |  |
|  |  |  |  |
|  |  |  |  |
|  |  |  |  |
|  |  |  |  |
|  |  |  |  |
|  |  |  |  |
|  |  |  |  |
|  |  |  |  |
|  |  |  |  |
|  |  |  |  |
|  |  |  |  |
|  |  |  |  |
|  |  |  |  |
|  |  |  |  |
|  |  |  |  |

**LIST OF FIGURES**

| 1 | **ACC main window** | p. | 5 |
| --- | --- | --- | --- |
| 2 | **Standard Screening Structure format specification** | p. | 6 |
| 3 | **GUI: load a dataset** | p. | 9 |
| 4 | **ACC main window (when a dataset is loaded)** | p. | 10 |
| 5 | **ACC main window: sections** | p. | 11 |
| 6 | **Menu and toolbar buttons** | p. | 12 |
| 7 | **Cell view window** | p. | 12 |
| 8 | **Image selector window** | p. | 13 |
| 9 | **File menu: list of buttons** | p. | 13 |
| 10 | **GUI: change settings** | p. | 15 |
| 11 | **Visualization menu: list of buttons** | p. | 15 |
| 12 | **Contour visualization** | p. | 16 |
| 13 | **Annotation menu: list of buttons** | p. | 17 |
| 14 | **GUI: create a new class** | p. | 18 |
| 15 | **Class icons and annotated cells** | p. | 19 |
| 16 | **Similar cells automatically detected** | p. | 20 |
| 17 | **GUI: find similar cells settings** | p. | 21 |
| 18 | **GUI: active learning settings** | p. | 22 |
| 19 | **Phenotype finder proof of concept** | p. | 23 |
| 20 | **GUI: phenotype finder settings** | p. | 24 |
| 21 | **Outlier tree** | p. | 25 |
| 22 | **Classification menu: list of buttons** | p. | 26 |
| 23 | **GUI: classification settings** | p. | 26 |
| 24 | **GUI: create report** | p. | 29 |
| 25 | **GUI: feature-based statistics settings** | p. | 32 |
| 26 | **GUI: CellProfiler "ExportToACC" module** | p. | 35 |
|  |  |  |  |

**1. BRIEF DESCRIPTION**

Advanced Cell Classifier (ACC) is a user friendly, data visualization and analyser software tool for cell-based high-content screens and tissue section images. The main aim of ACC is to provide accurate phenotypic analysis using advanced machine learning methods with minimal user interaction.

The project was started by Peter Horvath at ETH Zurich, Switzerland, in 2007. Now it is developed at the Biological Research Centre of the Hungarian Academy of Sciences in Szeged, Hungary, and at the Molecular Medicine Institute Finland (FIMM) in Helsinki. ACC was used to analyze some of the first whole-genome RNAi screens and for over 300.000.000 images and several billion single cell-based machine learning decisions to date.

This document is a short help tutorial to describe the main functions of ACC. It is written for non-experts. Additional information, video tutorials, source code, and literature references are available at: www.cellclassifier.org

**2. LICENSE**

The software and all the materials available at the www.cellclassifier.org website are copyright protected.

Copyright (©) 2016 Peter Horvath. All rights reserved.

Advanced Cell Classifier (ACC) is licensed under the:

GNU General Public License version 3

ACC is a free software: you can redistribute it and/or modify it under the terms of the GNU General Public License as published by the Free Software Foundation, either version 3 of the License, or any later versions (at your option).

This program is distributed in the hope that it will be useful, but WITHOUT ANY WARRANTY; without even the implied warranty of MERCHANTABILITY or FITNESS FOR A PARTICULAR PURPOSE. See the GNU General Public License for more details.

**3. SYSTEM REQUIREMENTS**

ACC is written in MATLAB (The MathWorks, Inc., Natick, MA, USA). It requires MATLAB R2015a, or any later version, and the Image Processing Toolbox 9.2. ACC works under Windows, Linux, and OS X environments.

**4. INSTALLATION**

1. Download the ACC source file from: www.cellclassifier.org
2. Extract the files from "ACC_v#.zip" to an arbitrary folder.
3. Open MATLAB.
4. Set the MATLAB's path to the ACC folder containing the *startup.m* file.
5. Type "startup" in the MATLAB Command Window.

Upon typing “startup”, the following window appears:

**Fig. 1**: ACC main window (before a project is opened/loaded).

**5. INPUT DATASET STRUCTURE**

To be able to open a dataset with ACC, it should be organized following the Standard Screening Structure (SSS) format specification, schematised in **Fig.2**. An SSS is a general data container designed for high content screening data such as image files and associated analyses. SSS can store an arbitrary number of data folders, however three folders, including composite images, segmented images and single cell-based metadata must be present for ACC. The latter is usually stored in simple text ".txt" files (hereafter it will be referred to as "SSS(.txt)"). However, other types of SSS can also be supported (e.g. "SSS(.h5)").

A sample dataset (*Test-ProjectFolder01.zip*) following the "SSS(.txt)" structure is available at the website: www.cellclassifier.org.

**Fig. 2**: SSS(.txt): txt-based Standard Screening Structure (SSS) format specification.

**PROJECT FOLDER, PLATE FOLDERS and DATA FOLDERS levels**

The SSS is based on a three-level folder structure, called "Project folder", "Plate folders", and "Data folders", respectively. The first level, "Project folder", is the main folder of the dataset. It may contain any subfolders (called "Plate folders"), each representing one plate of the dataset. Names of the "Plate folders" are internally used by ACC as the names of the plates. Each "Plate folder" must contain 3 subfolders, called "Contour Folder", "Metadata folder" and "Original image folder", respectively. Creators of ACC typically named the "Contour Folder" as *anal1*, "Metadata folder" as *anal2*, and "Original image folder" as *anal3.* However, the names *anal1*, *anal2* and *anal3* can be modified by the user when a "New project" is opened in ACC. The image types supported by ACC are: ".tif", ".bmp", ".png", ".jpg", and ".gif".

**CONTOUR FOLDER**

"Contour Folder" (*anal1*) contains segmented images. These are RGB images with contours highlighting individual objects. When sub-objects or subcellular compartments are visualized, we recommend using different colour combinations for their contours.

**METADATA FOLDER**

“Metadata folder” (*anal2*) contains metafiles (typically ".txt" files with numerical values separated by a white space) for each image. A datafile has as many rows as the number of the cells present in the image, and as many columns as the number of features computed for each cell. For instance, in case of an image containing three cells and four features, the metafile is written as follows:

1089.115801 20.78463203 924 1.009641262

1058.715994 58.76233184 669 1.007492525

1057.370014 96.97936726 727 1.014671698

The numerical values (separated by a white space) regard the different features extracted, typically computed by CellProfiler (http://cellprofiler.org/). The first two numbers always refer to the location of the cell in the image (i.e. *[x-column, y-row]*, with the origin *[0, 0]* at the upper-left corner of the image)*.* Besides, the *anal2* folder must contain a file named *featureNames.acc*, reporting the headers of the numerical values stored in the text files. The headers are listed in different rows (note that each header must be a single word with no white spaces allowed, e.g. *Nuclei_Location_Center_X*), for instance:

Nuclei_Location_Center_X

Nuclei_Location_Center_Y

Nuclei_AreaShape_Area

Nuclei_AreaShape_Compactness

**ORIGINAL IMAGE FOLDER**

“Original Image Folder” (*anal3*) contains the original RGB images. Typically, the original RGB images present in the Blue channel as a gray-level image referring to a nuclear staining (e.g. DAPI), in the Green channel as one referring to a GFP signal, and in the Red channel as an RFP, but it is absolutely arbitrary.

**GENERATING SSS structures**

We provide extension modules and sample pipelines for CellProfiler *v1.x* (written in MATLAB) and *v2.*x (written in Python) to create the SSS structure (see **Section 12. ACC and CellProfiler**).

**PLATE TYPES SUPPORTED by ACC and UNSTRUCTURED DATA**

ACC is designed to visualize and analyse images acquired by high-content screening tools. ACC currently supports the following multi-well "Plate types":

```
	1 	well 	(1 column x 1 row)
	6 	wells 	(3 columns x 2 rows)
	12 	wells 	(4 columns x 3 rows)
	24 	wells 	(6 columns x 4 rows)
	96 	wells 	(12 columns x 8 rows)
	384 	wells 	(24 columns x 16 rows)
	Unstructured data
```

It is worthy of note that in addition to the multi-well plate typologies, ACC may also accept *Unstructured data*. If *Unstructured data* are used, there is no need to follow the nomenclature described above as each image is considered as acquired in a separate well without a spatial location reference. New plate types can be easily added by modifying the "*ACC.ini"* file included in the *ACC "Utils" subfolder*.

**5.1 IMAGE NOMENCLATURE**

Image and metadata files have the following naming convention:

*PlateName_w%Row%Column_s%Num_*.ext*

*PlateName* is the name of the plate. It must be the same as the name of the "Plate folder" containing *anal1*, *anal2*, and *anal3*. The first character of *PlateName* must not be a number. Any characters are allowed to be used for the *PlateName*, except "_", "*" and white spaces. "*%Row*" is always preceded by "*_w*" (standing for well), and it is a digit from *A* to *H* in case of a 96-well plate, or from *A* to *P* in case of a 384-well plate (and a similar rule applies for the other types of multi-well plates supported by ACC). "*%Column*" is a number from *01* (please, note the *0* before the *1*) to *12* in case of a 96-well plate, or from *01* to *24* for a 384-well plate. It is worthy of note that numbers with a single digit are completed with '0' to make two characters. "*%Num*" is preceded by "*_s*" (standing for site), and it identifies the different individual images acquired in the same well. "***" is the remaining part of the name. This may contain channel information, time series information, and other. The "*.ext*" refers to file extension which is typically ".txt" for the files in *anal2*, or ".tif" for the files in *anal1* or *anal3*.

An example of file name is:

*Plate001_wB01_s03.tif*

where "*Plate001*" is the name of the plate, while "*B*" specifies the row and "*01*" specifies the column of the well. "*03*" is the number of the image. It is worthy of note that in this case the "Plate folder" containing *anal1*, *anal2*, and *anal3* must be named "*Plate001*", and the ".txt" file in *anal2* must be named as:

*Plate001_wB01_s03.txt*

**6. GETTING STARTED**

As a start, follow the steps described under **"Installation"** in **Section 4.** to install ACC to your computer. Then proceed as follows:

1. Download the example dataset (named *Test-ProjectFolder01.zip*) available at the website: www.cellclassifier.org.
2. Extract the files into a local folder of your computer and take a look at the structure of the folders. An example is described in **Section 5 (Input dataset structure).**
3. Take a look at the names of the files in the *anal#* folders. File names follow the structure described in **Section 5.1 (Image nomenclature).**
4. Open the ACC main window and click on: "File" -> "New project" to load the dataset. The intuitive windows reported in **Fig. 3** will appear to help the user in loading the dataset:

**Fig. 3**: GUI: load a dataset.

5. For "Dataset path" set the local path of the "Test-ProjectFolder01" folder. For "Plate type" select "*96 well (12x8)*". For the other fields keep the default values.

Upon clicking the “OK” button, the "Image selector" window appears, and the main window of ACC changes as follows:

**Fig. 4**: ACC main window when a dataset is loaded.

When a dataset is loaded, ACC is ready for the cellular analysis. The main steps for any follow-up analysis include:

1. Create classes of interest.
2. Annotate cells to show different phenotypes of interest.
3. Train a classifier to qualify it to automatically classify non-annotated cells.
4. Run the automatic classification to classify the cells of a specific image and to visually check the quality of the classification.
5. Repeat steps "2" and "3" to improve classification.
6. Classify the cells of the entire plate.
7. Save and output the data.

All these steps, as well as the functions implemented to create classes and annotate, visualize and analyse cells, are described in detail in the following sections. However, to give a short overview, we report an example of workflow here:

Example of a typical workflow:

1. Start ACC by typing "startup" in the MATLAB Command Window
2. Open a dataset with "File" -> "New project"
3. Improve image visualization using the "Visualization" functions available
4. Create classes with "Annotation" -> "Create a new class" (at least two of them)
5. Browse the images and annotate several cells for the defined classes by selecting a cell and then clicking on the corresponding class
6. Train a classifier with "Classification" -> "Classification settings"
7. Check the training quality with "Classification" -> "Predict current image": go through the images, run “Predict current image”, and correct cells classified improperly.
8. Classify all images with "Classification" -> "Predict selected plates"
9. Export feature-based statistics to an excel sheet with "Classification" -> "Feature-based statistics"
10. Save data with "File" -> "Save project"

**7. GRAPHICAL USER INTERFACE (GUI)**

When a new project is opened correctly (i.e. a dataset is loaded correctly), the first image of the first plate is shown in the ACC main window, and the first cell appears in the "Cell view" window on the right side of the ACC main window.

**Fig. 5**: ACC main window: sections.

All the ACC commands are activated by using the menu items. The most common commands can also be activated by using toolbar icons. To help the user to understand the functionality of the different buttons, short help messages appear once leaving the mouse on the buttons.

**Fig. 6**: Menu and toolbar buttons.

**7.1 CELL VIEW**

The first cell, i.e. the first image of the first plate, is automatically visualized in the "Cell view" window which is located on the right side of the main window. The Red, Green and Blue channels of the selected cell are separately shown on different panels. Furthermore, the first panel shows the merged colour image. The intensity of signals visualized in the "Cell view" window may be modified in the "Visualization" menu.

**Fig. 7**: "Cell view" window with panels for visualizing the Red, Green and Blue channels of the selected cell.

**7.2 IMAGE SELECTOR**

The "Image selector" window automatically appears when a dataset is loaded correctly. It cannot be closed by the user and it is automatically updated when plates are added or removed, or when a different dataset is loaded. "Image selector" allows the user to select the image to be visualized in the "Main view" of ACC.

**Fig. 8**: "Image selector" window to select the image to be visualized in the main window of ACC.

**8. FILE MENU**

**Fig. 9** shows the list of buttons included in the "File" menu.

**Fig. 9**: File menu: list of buttons.

**8.1 NEW, OPEN, AND SAVE PROJECT**

To start a new project click "New project": the window shown in **Fig. 3** appears. User may define the path to the "Project folder" of the dataset, the names of "Contour folder", "Metadata folder", and "Original image folder" (see **Section 5. Input dataset structure**), the plate typology, the extension of the images in the "Contour folder" and "Original image folder", and the SSS of the dataset to be loaded. When "Open project" button is pressed, a basic window appears to simply select the path of a previously saved project (a saved project is a ".mat" file). Similarly, when "Save project" button is clicked, another window appears to simply define the path for the project to be saved. Previously created classes and annotated cells are automatically saved within a project file. Furthermore, it is worthy of note that "New project" and "Open project" buttons may be clicked anytime during the ACC execution. In case of loading a dataset/project remember to save the current classes, otherwise they are automatically deleted. However, once a dataset is loaded correctly and the "New project" button is pressed accidentally, no problematic changes appear (except that the first image of the first plate is automatically shown in the "Main window").

**8.2 ADD AND REMOVE PLATES**

"Add plates" and "Remove plates" buttons allow the user to select which plates to add to or exclude from the "Project folder".

**8.3 IMPORT TRAINED DATA AND CHANGE SETTINGS**

The "Import trained data" button allows the user to import annotated cells from a previously saved project (i.e. a ".mat" file). When "Import trained data" button is pressed, a window appears to simply select the path of the saved project. Instead, when "Change settings" button is pressed, the window shown in **Fig. 10** appears. It allows changing the "Project folder" path, the names of the "Contour folder", "Metadata folder" and "Original image folder", as well as the plate typology of the dataset. This command is useful when the dataset is moved to another hard disk, for example.

**Fig. 10**: GUI: change settings.

**9. VISUALIZATION**

**Fig. 11** shows the list of buttons of the "Visualization" menu.

**Fig. 11**: Visualization menu: list of buttons.

**CELL NUMBERS (ON/OFF)**

"Cell numbers" button enables/disables the visualization of the Identification Number (ID) of the cells in the "Main window". The first cell visualized in each image is always marked as ID 1 (to check this, simply change the image displayed using the "Image selector" window). The functionality of this button changes after having performed a classification on the current image (see the description for the "Predict current image" button in the "Classification" menu). If the "Predict current image" command is successfully run, the cell numbers turn to a number indicating the cell's phenotypic class. To restore the standard visualization of "Cell numbers", simply change the visualized image using the "Image selector" window.

**CELL CLASSES (ON/OFF)**

Each manually annotated cell is associated with one of the available classes, and is identified by an ordinal number of that associated class accordingly. "Cell classes" button enables/disables the visualization of the number indicating the class of the annotated cells.

**CONTOURS (ON/OFF)**

"Contours" button selects the visualization of the contours (lines separating different cells) saved as saturated pixels in the images of the "Contour folder". "Contours" button modifies the visualization of the image shown in the main window of ACC.

**Fig. 12**: Contour visualization.

**RED, GREEN, and BLUE CHANNEL (ON/OFF)**

"Red channel", "Green channel", and "Blue channel" buttons allow to enable/disable the visualization of individual fluorescence channels.

**IMAGE COLOURS (ON/OFF)**

"Image colours" button allows the user to change the visualization of the images between colours and monochromatic gray-levels. Cases/problems are often easier to be examined, or look more natural when the cells are visualized in gray-scale mode.

**STRETCH IMAGE INTENSITY (ON/OFF) and IMAGE COLOUR SETTINGS**

Both "Stretch image intensity" and "Image colour settings" buttons help the user to improve the visualization of the images. The "Stretch image intensity" button automatically increases the contrast of the image, while the "Image colour settings" button opens a GUI allowing the user to manually stretch individual channels of the image.

**10. ANNOTATION**

**Fig. 13** shows the list of buttons included in the "Annotation" menu.

**Fig. 13**: Annotation menu: list of buttons.

**10.1 CREATE, DELETE, LOAD, AND SAVE CLASSES**

This group of buttons allow the user to manage classes such as to create a new phenotypic class, delete an existing class, load previously saved classes, and save the current one as a ".mat" file. It is worthy to note that "Load classes" and "Save current classes" buttons serve to load and save the classe only, but they do not save the annotated cells. Accordingly, if you (*a*) save classes containing some annotated cells and then (*b*) delete all the classes, whenever you (*c*) load back the saved ones, (*d*) the classes will appear with no annotated cell. To create a class simply click on the "Create a new class" button. The GUI shown in **Fig. 14** appears.

**Fig. 14**: GUI: create a new class.

The user must define a "Class name" that has not been used for other classes before. By default, the last selected cell is automatically used as icon of the new class. In case of choosing the "Normal" class type, subclasses (called "Child"-classes) may also be created. Icons of all classes created automatically appear at the bottom of the "Main window". Upon pressing the blue rectangular button at the bottom-left-corner of all icons of a class, a list of annotated cells is visualized as shown in **Fig. 15**.

**Fig. 15**: Class icons and annotated cells.

**10.2 MANUAL ANNOTATION**

The basic modality to annotate a cell with a class label is manual annotation. To manually annotate a cell:

1. Click on the cell you want to annotate.
2. Click on the icon of the class.

However, 4 other annotation modalities are also available in ACC, including:

- Random annotation
- Find similar cells
- Active learning
- Phenotype finder

**10.3 RANDOM ANNOTATION**

When "Random annotation" button is ON, cells are randomly sampled from the entire dataset and are displayed to the user. After annotation of one cell, the next randomly selected cell is automatically displayed. The user may always choose to select and annotate another cell, different from the randomly displayed one. To finish the annotation process, switch the "Random annotation" button OFF. This procedure may also be helpful in finding new classes of interest and to create an unbiased sampling set.

**10.4 FIND SIMILAR CELL**

"Find similar cells" button helps the user to find cells similar to the currently selected one. This function may be really useful to increase the number of annotated cells within a class. Furthermore, it can help to find cells similar to a rarely appearing cell type with a phenotype of interest. To use find similar cells:

1. Manually select a cell of interest.
2. Click on the "Find similar cells" button.

A list of similar cells, automatically detected, will appear (see **Fig. 16**). If you want to include any of those cells in the class, you can use the "Jump to cell" button and then annotate the cell.

**Fig. 16**: Similar cells automatically detected.

Parameters of the "Find similar cells" method are shown in **Fig. 17**.

**Fig. 17**: GUI: Find similar cells settings.

- **Max number of cells** (value in the range of *[1, 200]*): the maximum number of similar cells shown after clicking the "Find similar cells (run)" button.
- **Min similarity score** (value in the range of *[0, 1]*): the parameter that defines the extent of "similarity required" for the cells displayed as similar ones. If "Min similarity score" is set around 1, then few cells are shown as "similar cells". Typically, values in the range of 0.4–0.8 are used.
- **Max feature similarity** (value in the range of *[0, 1]*): if the similarity of two features examined exceed the "Max feature similarity" setting value, one of the features is then not used in the "Find similar cells" search. Value 1.0 keeps all the features, except identical ones. Value 0.0 removes all but one feature. Typically, values in the range of 0.90-0.95 are used.
- **Feature variance** (value in the range of *[0, 1]*): the features are ranked based on their variance (after normalization) and the "Feature variance" setting serves to select the features to be used in the "Find similar cells" search. All features with a normalized variance lower than the "Feature variance" value are excluded. Value 0.0 keeps all features. Value 1.0 removes all but one feature. Typically, values in the range of 0.4–0.8 are used.

**10.5 ACTIVE LEARNING**

The general idea of "Active learning" algorithms is to help the user to improve the decision ability of the machine learning model by on-line selection of the minimal set of cells to be annotated. In practice, "Active learning" algorithms first automatically select cells that are difficult to classify, then ask the user to manually annotate those cells. This process results in a better boundary definition between the classes.

To use "Active learning" efficiently, the user must:

1. Manually define the classes of interest (at least two).
2. Manually annotate a starting set of representative cells (a minimum of 5 cells per class). The “Random selection” strategy is used until this minimal number is reached.

Different "Active learning" strategies have been proposed in the literature. For a better overview we suggest reading the article: "Smith K, Horvath P (2014). Active learning strategies for phenotypic profiling of high-content screens. Journal of Biomolecular Screening". The current document is not intended to describe the variety of "Active learning" strategies, neither to explain how to set the specific parameters of each algorithm. We remark that the best "Active learning" strategy is dataset dependent (see *Smith and Horvath, 2014*). Accordingly, here we list only the "Active learning" algorithms currently implemented in ACC:

- Committee members
- Uncertainty sampling

For non-computer experts we recommend using the "Committee members" algorithm, where setting the "Size of committee" parameter to 3 generally provides good results.

**Fig. 18**: GUI: active learning settings.

**10.6 PHENOTYPE FINDER**

Another typical and absolutely crucial data exploration task is identifying cell types which have never been seen by the user before. "Phenotype finder" refers to a weakly-supervised machine learning algorithm module, useful to automatically analyse the dataset looking for special cells most dissimilar to those annotated in the available classes. Practically, starting from the annotated cells (green, blue and yellow regions in **Fig. 19**), "Phenotype finder" first defines the space of non-annotated cells (red region) to find the cells that are the least similar to those annotated. As a second step, analysing the "outlier" cells, the algorithm builds a hierarchical tree trying to identify sub-groups of similar phenotypes. This may help the user to find new classes of interest.

**Fig. 19**: Phenotype finder proof of concept.

"Phenotype finder" requires several settings. Then, when "Phenotype finder settings" button is clicked, the window shown in **Fig. 20** appears.

**Fig. 20**: GUI: phenotype finder settings.

"Number of selected cells" means the number of cells shown in the hierarchical tree panel. It is worthy to note that the user can "Jump to cell" to see the selected cell in the image and visually check its context before creating a new class. "Metric" and "Method" refer to the internal strategy used by the semi-supervised machine algorithm to define the hierarchical tree. "Final number of attributes" means the number of features considered to build the tree of sub-classes. No general optimal parameter configuration exists because the settings are dataset dependent. However, we suggest to use the option "calculate the best metric-method pair" and to set the final number of attributes to a value between 10–15.

Clicking on the "Phenotype finder run" button, the outlier tree shown in **Fig. 21** will appear. Clicking on a class icon one can subdivide the cells of the class into sub-classes. The red rectangular button on the bottom-right-corner of each icon allows the user to directly define a new class and insert all the underlying cells. The green rectangular button visualizes cells that are included in the current sub-tree.

**Fig. 21**: Outlier tree.

**10.7 GENERATE SAMPLING POOL**

"Find similar cells", "Active learning", and "Phenotype finder" are tools that automatically analyse the dataset to help the user to better define classes and annotated cells. However, scanning the entire dataset is a very time-consuming procedure. "Generate sampling pool" allows to reduce the dataset of cells to be considered sufficient for the analyses. It is important to note, however, that these processes require to read the entire data several times, therefore it is highly desired to have a memory portion as large as possible. When "Generate sampling pool" button is clicked, a setting window appears asking the user for the "desired sampling ratio", that is a number *x*, where *0* < *x* ≤ *1* represents the percentage of the original dataset to be kept for further consideration. Accordingly, if "desired sampling ratio" is set to *1* the entire original dataset is used.

**11. CLASSIFICATION**

**Fig. 22** shows the list of buttons included in the "Classification" menu.

**Fig. 22**: Classification menu: list of buttons.

**11.1 CLASSIFICATION SETTINGS**

When "Classification settings" button is clicked, the window shown in **Fig. 23** appears.

**Fig. 23**: GUI: classification settings.

Currently, 16 different classifiers are available:

1. Multi Layer Perceptron (*MLP_Weka*) – also known as Artificial Neural Networks. This is the #1 recommendation of the Authors. It is especially suitable for difficult-to-recognize phenotypes. Often a larger number of annotated cells is recommended (50-500 cells per class).
2. Random Forest (*RandomForest_Weka*) – Random Forest classifiers have very strong generalization abilities. It is a very powerful tool to distinguish between rather simple phenotypes. Two major advantages we have observed are that a low number of training samples are sufficient and its very short training time makes this classifier convenient to be used for quick discoveries.
3. Logistic Boost (*LogitBoost_Weka*) – With no intention to explain the theory behind this classifier, in practice it shows highly desirable properties. We would position this method in between the former two as a fairly fast and very precise method.
4. Adaboost (*Adaboost_Weka*)
5. Bayes Network (*BayesNet_Weka*)
6. Boosted Stump (*BoostedStump_Weka*)
7. Decision Tree (*DecisionTree_C45_Weka*)
8. Decision Table (*DecisionTable_Weka*)
9. K-Nearest Neighbours (*KNN_Weka*)
10. K*(*KStar_Weka*)
11. Logistic Regression (*LogisticRegression_Weka*)
12. Naïve Bayes classifier (*NaiveBayes_Weka*) – usually used as a baseline classifier. It is often claimed that if other classifiers cannot outperform this one, than the problem is too hard to learn or the used feature-set is non-descriptive.
13. Nearest Neighbor (*NearestNeighbor_Weka*)
14. Random Tree (*RandomTree_Weka*)
15. Support Vector Machine (*SVM_Libsvm*)
16. Support Vector Machine (*SVM_Weka*) – classical SVM. With proper parameter settings it is very fast and often precise.

Regadring the scope of this document, we do not intend to explain the different classification algorithms. However, for non-computer scientists we propose to choose multilayer perceptron (i.e. MLP_Weka), which generally provides very good results. Furthermore, there is no general rule for an efficient choice of a classifier. To train the selected classifier simply click "Train" button in **Fig. 22**.

**12. OUTPUT**

ACC is a user friendly software to characterize the cell phenotypes present in the cell culture analysed. The main data obtained as output from ACC include:

1. Cell-by-cell classification.
2. Incidence and distribution of the different classes.
3. Class-based statistics for the different features computed.

The classification of each cell on the plate set can be checked, either by directly displaying the image generated by the "Predict current image" command, or by reading the file "*PlateName*_singleCellData.csv" generated by the "Predict selected plates" command. To gain quantitative data about the incidence and distribution of the different classes check the "*PlateName*.csv", "*PlateName*_cumul_*%Sel*_v_*%Norm*.csv", and "*PlateName*_cumul_*%Sel*_v_*%Norm*.pdf" files generated by the "Predict selected plates" command. Finally, the file "*PlateName_fb.csv*", generated by the "Feature-based statistics" command reports statistics on the different features aggregated well-level or class-level. The following subsections provide more details on how to generate the output results.

**12.1 PREDICT CURRENT IMAGE**

The "Predict current image" button classifies the cells of the current image. However, it is important that a classifier must be trained before running the "Predict current image" button. To use this function for visually checking how a classifier performs, follow these steps:

1. Create at least two classes of interest.
2. Annotate a few manually selected cells for each class.
3. Train the classifier by clicking on the "Classification settings" button.
4. Select an image of interest.
5. Set the "Cell numbers" button ON.
6. Press the "Predict current image" button.

Identification numbers, corresponding to the ordinal number of the available classes, will be automatically shown on the cells, indicating the class where non-manually annotated cells have been classified into by the trained classifier.

**12.2 PREDICT SELECTED PLATES**

"Predict selected plates" button generates statistics on the cells. Its precondition is that a trained classifier exists, i.e. some classes must be predefined and must contain annotated cells.

When the "Predict selected plates" button is pressed, the window shown in **Fig. 24** appears.

**Fig. 24**: GUI: create report.

The field "Choose plates" allows the user to select which plates to be included in the statistical report. "Phenotypes for statistics" points out the classes of interest to be considered in the analysis. "Phenotypes for normalization" points out the classes to be used as the normalization factor. "Single cell classification result" enables/disables the creation of a file (i.e. "*PlateName*_singleCellData.csv") reporting the class of each cell in the dataset. Upon pressing "Start", several report files are automatically created and saved in the metadata folder of the plates selected in the field "Chose plates".

The main report files automatically saved to the metadata folder after pressing the "Start" button include:

1. *PlateName*.csv
2. *PlateName_*cellnumber.csv
3. *PlateName_*singleCellData.csv
4. *PlateName_*cumul_*%Sel*_v_*%Norm*.csv
5. *PlateName_*cumul_*%Sel*_v_*%Norm*.pdf

*PlateName* is the name of the plate. It must be the same as the name given to "Plate folder" containing *anal1*, *anal2*, and *anal3.* "*%Sel*" (standing for selected) is always preceded by "*_*", and it lists the classes (expressed with ordinal numbers) selected in the field "Phenotypes for statistics" (see **Fig. 22**). "*%Norm*" is always preceded by "*_*", and lists the ordinal numbers of the classes selected in the field "Phenotypes for normalization". For instance, upon pressing "Start" after setting the parameters as shown in **Fig. 22**, the real names of the files automatically saved in *anal2* are:

1. Test-PlateFolder01.csv
2. Test-PlateFolder01_cellnumber.csv
3. Test-PlateFolder01*_*singleCellData.csv
4. Test-PlateFolder01_cumul_1_v_1_2.csv
5. Test-PlateFolder01_cumul_1_v_1_2.pdf

**DESCRIPTION OF THE "*PlateName*.csv" FILE**

"*PlateName*.csv" is a comma-separated values (CSV) file. For each well of the multi-well plate analysed, "*PlateName*.csv" reports the following data in columns:

1. *PlateName*
2. *%Row*
3. *%Column*
4. Total number of cells analysed in the different wells
5. Hit rate
6. Number of cells classified in the "%*Norm*" classes

It is worthy to note that when more images of the same well are present (i.e. different sites) the values of these images are listed to make a single line for each well of the multi-well plate analysed. Hit rate is the ratio of the number of cells of the "*%Sel*" classes and the number of cells of the "*%Norm*" classes. A couple of rows of the "Test-PlateFolder01.csv" are shown below to give an overview of its content:

```
	PlateName,		Row,	Col,	ObjectNumber,	MainHitrate,	big,	small
	Test-PlateFolder01,	B,	4,	173,		0.468208,	81,	92
	Test-PlateFolder01,	C,	4,	211,		0.962085,	203,	8
```

**DESCRIPTION OF THE "*PlateName*_cellnumber.csv" FILE**

"*PlateName*_cellnumber.csv" shows the total number of cells analysed in the different wells of the multi-well plate.

**DESCRIPTION OF THE "*PlateName*_singleCellData.csv" FILE**

"*PlateName*_singleCellData.csv" is automatically saved to the metadata folder only when "Single cell classification result" is set ON. "*PlateName*_singleCellData.csv" is a comma-separated values (CSV) file. For each cell of the multi-well plate, "*PlateName*_singleCellData.csv" reports the following data in columns:

1. *PlateName*
2. *%Row*
3. *%Column*
4. Image name
5. Image number (i.e. exact site for multiple images coming from the same well)
6. *x*-coordinate of the centroid of the cells in pixels
7. *y*-coordinate of the centroid of the cells in pixels
8. cell class

A couple of rows of the "Test-PlateFolder01_singleCellData.csv" are shown below to give an overview of its content:

PlateName, Row, Col, ImageName, ImageNumber, ObjectNumber, xPixelPos, yPixelPos, Class

Test-PlateFolder02, B, 4, Test-PlateFolder02_wB04_s01_t01, 1, 20, 369.272450, 533.645820, 2

Test-PlateFolder02, B, 4, Test-PlateFolder02_wB04_s01_t01, 1, 21, 405.294020, 475.501650, 1

**DESCRIPTION OF THE "*PlateName*_cumul_*%Sel*_v_*%Norm*.csv" FILE**

"*PlateName_*cumul_*%Sel_*v_*%Norm*.csv" shows the hit rate of the cells classified as belonging to one of the "*%Sel*" classes, normalized with the number of cells classified as belonging to one of the "*%Norm*" classes.

**DESCRIPTION OF THE "*PlateName*_cumul_*%Sel*_v_*%Norm*.pdf" FILE**

"*PlateName_*cumul_*%Sel_*v_*%Norm*.pdf" is a ".pdf" file reporting the following data in colour scale bars: the hit rate and the number of cells classified as belonging to one of the "*%Sel*" classes, normalized with the number of cells classified as belonging to one of the "*%Norm*" classes. Furthermore, hit rate and cell number values are also presented as plate-based row-wise and column-wise average.

**12.3 FEATURE-BASED STATISTICS**

The "Feature-based statistics" button generates statistics for the different features aggregated well-level or class-level (e.g. average signal intensity of the cells classified as mitotic). Please note that to run "Feature-based statistics" in a class-level mode, some classes must be predefined and they must contain annotated cells, a trained classifier should exist, and the current plate must have been previously predicted by using the "Predict selected plate" button.

When the "Feature-based statistics" button is pressed, the window shown in **Fig. 25** appears.

**Fig. 25**: GUI: feature-based statistics settings.

The field "Choose plates" allows the user to select the plates, and the field "Export features" allows to select the features to be included in the statistical report.

Choosing "Feature based" in the "Statistic type", two additional parameters appear: "Detailed statistics" and "Class specific statistics". If "Detailed statistics" is set ON, the mean, standard deviation (i.e. std), median, and median absolute deviation (i.e. mad) values are computed for each selected feature. Otherwise, only mean values are calculated. If "Class specific statistics" is set ON, the feature-specific indices are computed well-level and class-level. Otherwise, only well-wise statistics are calculated.

Besides, the correlation coefficients for each pair of selected features are computed. The correlation coefficient is calculated considering all the cells in a well.

The files automatically saved to the metadata folder after pressing the "Export" button include:

- *PlateName_fb.csv* (when "Feature based" is set ON)
- *PlateName_ccb.csv* (when "Correlation based" is set ON)

**DESCRIPTION OF THE "*PlateName_fb.csv* " FILE**

"*PlateName_fb.csv*" is automatically saved to the metadata folder only when "Feature based" is set ON. "*PlateName_fb.csv*" is a comma-separated values (CSV) file. For each cell of the multi-well plate, "*PlateName_fb.csv*" reports the total number of cells and the indices computed for the selected features, in columns.

**DESCRIPTION OF THE "*PlateName_ccb.csv* " FILE**

"*PlateName_ccb.csv*" is automatically saved to the metadata folder only when "Correlation based" is set ON. "*PlateName_ccb.csv*" is a comma-separated values (CSV) file. For each cell of the multi-well plate, "*PlateName_ccb.csv*" reports the total number of cells and the correlation coefficient computed for each pair of selected features, in columns. The names of the columns inform the reader about the features used for computing the correlation coefficient. For instance, the output file column "f3_vs_f7" reports the correlation coefficient computed for features No. 3 and No. 7.

**13. CAMIO - CELL UPLOADING MODULE**

v2.1 contains an uploader module to interact with CAMIO databases. This module allows user to upload single-cells that belong to a certain phenotype of interest.

Before running this uploading module, it is required to have a trained classifier.

By clicking the CAMIO button on the toolbar, a window will pop-up. User may navigate between existing classes (left side); set the number of the representative cells to be uploaded (right side).

Using the "Network" button, user can set up the hostname, the port and the path where the cells will be uploaded. With the "Directory" button, the user can set up the name of the specific folders (cell and nuclei segmentation), the path for the descriptor files and the path for the image file that contains the marker.

After selecting the phenotypic class and setting the number of the interesting cells; pressing "Show cells" button will open a new window with a list of cells.	A preselection can be made and single-cell level comments can be added.

**14. ACC AND CELLPROFILER**

CellProfiler (http://cellprofiler.org/) is a widely used open-source software to obtain the input data needed for running ACC. CellProfiler is suitable to analyse images typically acquired by wide-field fluorescence microscopes. CellProfiler allows the user to easily create the RGB images to be stored in the "Contour Folder" (*anal1*) and in the "Original image folder" (*anal3*).

The RGB images in the "Original image folder" are obtained simply by merging the different fluorescent channels and rescaling the intensity. Hereunder an example of CellProfiler pipeline to obtain these RGB images is presented (here we report only the names of the main modules):

```
	'LoadImages'		-> To load the original mono-channel grey-level images
	'RescaleIntensity'	-> To rescale the intensity of the first channel
	'RescaleIntensity'	-> To rescale the intensity of the second channel
	'RescaleIntensity'	-> To rescale the intensity of the third channel
	'GrayToColor'		-> To merge the three mono-channel grey-level images, 					   intensity rescaled, in a single RGB image
	'SaveImages'		-> To save the final RGB images in anal3
```

Similarly, the RGB images to be stored in the "Contour Folder" can be obtained by overlaying contours in the "Original image folder" images. We suggest using different colours for the contours and their related objects, because upon using the same colour for representing the object's contour and the object itself, the line of the contour is not obviously visible. As the nucleous is typically reported in blue, and the cytoplasm is typically reported in green, we suggest to use green colour (Green channel) for representing the contour of the nucleous and blue (Blue channel) for the contour of the cytoplasm:

```
	'LoadImages'			-> To load the original mono-channel grey-level images
	'RescaleIntensity'		-> To rescale the intensity of the first channel
	'RescaleIntensity'		-> To rescale the intensity of the second channel
	'RescaleIntensity'		-> To rescale the intensity of the third channel
	'IdentifyPrimAutomatic'	-> To segment the nuclei
	'IdentifySecondary'		-> To segment the cells
	'OverlayOutlines'		-> To overlay the nuclei contours in the green channel
	'OverlayOutlines'		-> To overlay the cell contours in the blue channel
	'GrayToColor'			-> To merge the three mono-channel grey-level images, 					   with contours, in a single RGB image
	'SaveImages'			-> To save the final RGB images in anal1
```

Finally, the ".txt" files required to be present in the "Metadata" folder may be obtained first by computing the feature measurements with CellProfiler, and then exporting the data to the ACC format. To export the data from CellProfiler to ACC we have developed the "ExportToACC" module. It works with CellProfiler *1.X* (MATLAB version) and *2.X* (Python version) and it is freely available at www.cellclassifier.org. Hereunder an example of CellProfiler's pipeline to create the ".txt" files required to be present in *anal2* is presented:

```
	'LoadImages'		   	-> To load the original mono-channel grey-level images
	'RescaleIntensity'	   	-> To rescale the intensity of the first channel
	'RescaleIntensity'	   	-> To rescale the intensity of the second channel
	'RescaleIntensity'	   	-> To rescale the intensity of the third channel
	'IdentifyPrimAutomatic'	-> To segment the nuclei
	'IdentifySecondary'	   	-> To segment the cells
	'IdentifyTertiarySubregion'   -> To segment the cytoplasm
	'OverlayOutlines'	   	-> To overlay the nuclei contours in the green channel
	'OverlayOutlines'	   	-> To overlay the cell contours in the blue channel
	'MeasureObjectIntensity'	-> To measure intensity features in the first channel
	'MeasureObjectIntensity' 	-> To measure intensity features in the second channel
	'MeasureObjectIntensity' 	-> To measure intensity features in the third channel
	'MeasureObjectAreaShape' 	-> To measure nucleus, cytoplasm, and cell 							   morphological features
	'MeasureTexture'	   	-> To measure texture features in the first channel
	'MeasureTexture'	   	-> To measure texture features in the second channel
	'MeasureTexture'	   	-> To measure texture features in the third channel
	'ExportToACC'		   	-> To save the ".txt" files in anal2
```

Finally, **Fig. 26** shows the GUI of the "ExportToACC" module.

**Fig. 26**: GUI: CellProfiler "ExportToACC" module.
