# Supplementary material for: Intelligent image-based in situ single-cell isolation
Source: Nat Commun. 2018 Jan 15;9:226. doi: 10.1038/s41467-017-02628-4 (PMC5768687; doi:10.1038/s41467-017-02628-4)
Supplement: Supplementary file 7 — Supplementary Software [file 41467_2017_2628_MOESM7_ESM.zip › SuppSW4_CAMIO_Book.zip!/CAMIO User Manual.docx]

CAMIO - user manual
http://camio-webapp.herokuapps.com

**v1.0 - Nov 2016**
(written by Csaba Molnar)

**Prof. Peter Horvath, PhD**
Synthetic and Systems Biology Unit,
Biological Research Center of the
Hungarian Academy of Sciences
[horvath.peter@brc.mta.hu](mailto:horvath.peter@brc.mta.hu)

INDEX

[1. BRIEF DESCRIPTION	4](#_Toc489588751)

[2. REQUIREMENTS	4](#_Toc489588752)

[JAVA RUNTIME ENVIRONMENT (JRE)	4](#_Toc489588753)

[3. INSTALLATION	5](#_Toc489588754)

[This section gives a brief guide to CAMIO installation.	5](#_Toc489588755)

[3.1 INITIALIZE MYSQL DATABASE	5](#_Toc489588756)

[3.2 DEPLOYING WEB APPLICATION	6](#_Toc489588757)

[DEPLOYING ON SERVER	6](#_Toc489588758)

[RUNNING APPLICATION	6](#_Toc489588759)

[4. GETTING STARTED	7](#_Toc489588760)

[4.1 BROWSING DATA	7](#_Toc489588761)

[SCREEN VIEW	7](#_Toc489588762)

[EXPERIMENT VIEW	9](#_Toc489588763)

[CELL VIEW	10](#_Toc489588764)

[IMAGE VIEW	11](#_Toc489588765)

[4.2 EDITING DATA	12](#_Toc489588766)

[EDITING CELL CONTOURS	12](#_Toc489588767)

[4.3 EXPORTING DATA	12](#_Toc489588768)

[EXPORTING CELL CONTOURS	13](#_Toc489588769)

[(EXPORTING PDF)	13](#_Toc489588770)

| 1 | **Relational model of CAMIO database** | p. | 5 |
| --- | --- | --- | --- |
| 2 | **Screen view** | p. | 8 |
| 3 | **Experiment view** | p. | 9 |
| 4 | **Cell view** | p. | 10 |
| 5 | **Image view** | p. | 11 |
| 6 | **Cell view with contour edition** | p. | 12 |

**LIST OF FIGURES**

# 1. BRIEF DESCRIPTION

CAMIO is an interactive web-based cell database. Every single cell included in the database is selected by a supervised machine learning algorithm guided by a field expert’s annotation process (see [Advanced Cell Classifier](http://www.cellclassifier.org) by Horvath et al.).

# 2. REQUIREMENTS

CAMIO is a Java Spring based Maven project that works on Windows, Linux and OS X operating systems.

## JAVA RUNTIME ENVIRONMENT (JRE)

To run the application JRE 1.7 or a later version is required. You can download it [here](http://www.oracle.com/technetwork/java/javase/downloads/index.html).

**MYSQL**

The implementation of the database model relies on a MySQL framework. To run CAMIO you need to have access to a MySQL database, or you may have it installed on your local computer (see installation guide for [Ubuntu](https://help.ubuntu.com/12.04/serverguide/mysql.html) and for [Windows](http://dev.mysql.com/doc/refman/5.7/en/windows-installation.html)).

**APACHE TOMCAT**

Install [Apache Tomcat 8](http://tomcat.apache.org/tomcat-8.0-doc/index.html) as the Java web server onto your computer. Set environmental variable $CATALINA_HOME$ to install folder of Apache Tomcat.

# 3. INSTALLATION

## This section gives a brief guide to CAMIO installation.

## 3.1 INITIALIZE MYSQL DATABASE

We are using the following database model:

- ‘physical_experiment’ represents a project of a high-throughput screening microscope;
- ‘image’ represents a merged image of a field of interest;
- ‘logical_experiment’ represents a machine learning assisted decision on the most likely representatives of a phenotype;
- ‘cell’ represents a segmented cell of a logical experiment.

To create the database model, you need to run/import the **camio_initial_table_structure_V1.00.00.sql** SQL script. The script creates the empty tables of the relational model presented in **Figure 1**. Warning: take care that it drops the existing schema having the same name ”camio”.

Figure 1. Relational model of CAMIO database.

## 3.2 DEPLOYING WEB APPLICATION

### DEPLOYING ON SERVER

1. Download the **camio-webapp.war** file from the web page.
2. Extract **camio-webapp.war** file to the **$CATALINA_HOME$/webapps/camio-webapp** folder.
3. Set database and local media properties in **$CATALINA_HOME$/webapps/camio-webapp/WEB-INF/classes/camio.properties** config file in the format **variableName=value**.
   a. The required variables should be set to build up the database connection:
      **database.host**: the host and port of MySQL server in the format host:port, default values are **localhost:3306**;
      **database.path**: the name of database schema that contains the required tables; default (respectively to SQL script) is **/camio**;
      **database.user**: username to the database, default value is **root**;
      **database.password**: password of database user, default value is **rootpwd**;
   b. the path to access stored image files are defined by the following properties: **data.root.path**: path of saved media files (i.e. images), default value is **data/**;
      **image.file.path**: regular expression that describes the path of stored images of a screen; by default it starts with the value of data.root.path variable, and the default value is **data/physical_experiments/{physicalExperimentID}/{physicalExperimentName}/{colourDir}/{imageFileName}**;
   c. Two different storage modes are implemented in CAMIO: you can save your image files on your local computer or you can store them in Dropbox cloud. To store the images on your local computer, the following properties should be set:
      **file.storage.mode=file**;
      **file.storage.place**: root folder of image data, e.g. ’D:/camio/’;
      note that **cloud.dropbox.accesstoken** property should be set to empty string.
      To use the Dropbox cloud storage option you need to create an app [here](https://www.dropbox.com/developers/apps), [generate an access token](https://blogs.dropbox.com/developers/2014/05/generate-an-access-token-for-your-own-account/), and set the following properties:
      **file.storage.mode=cloud**;
      **file.storage.place**: name of cloud provider, CAMIO works with ’**dropbox**’ only;
      **cloud.dropbox.accesstoken**: access token generated to your Dropbox app.

## RUNNING APPLICATION

Restart Apache Tomcat server. You can find the running application at URL <http://localhost:8080/camio-webapp>.

# 4. GETTING STARTED

This section presents a brief overview on website usage.

## 4.1 BROWSING DATA

In CAMIO you can browse the experiments in different views:

- ‘screen’: a “physical experiment” containing the images and corresponding metadata;
- ‘experiment’: a “logical experiment” containing the cells of interest selected by a supervised machine learning algorithm;
- ‘image’: RGB color image of a field of interest;
- ‘cell’: single cell in a “logical experiment”.

Below we give a concise description of the contents of each view.

### SCREEN VIEW

A high-throughput microscopy project, i.e. the acquired images taken of our region of interest, is called ‘screen’. In ‘screen view’ properties of the screen (e.g. pixel size, marker position) with related experiments and the adjusted merged images in RGB color mode are shown (see **Figure 2**).

Figure 2. Example of a screen view

### EXPERIMENT VIEW

The term ‘experiment’ refers to a set of predicted and uploaded cells that are the most representative entities of a selected phenotype. In the ‘experiment view’ description of the experiment may be changed. Each cell can be visualized separately in a new browser tab (see ’cell view’ below). To export the contours of the cells to cut them out via microdissection microscope we can select those of interest by clicking on them. The selected cell is marked by a red rectangle around the thumbnail image of the cell. After selecting candidate cells the contour text file can be exported by clicking on the “Generate contours of selected cells” button (**Figure 3**).

Figure3. Example of an experiment view

### CELL VIEW

‘Cell view’ offers a detailed view of each single cell. Further details may be added to each cell using the input fields. Also, contours of the cells may be exported separately (**Figure 4**).

**Figure 4.** Example of a cell view

### IMAGE VIEW

Each field of interest can be visualized in RGB color mode (**Figure 5**).

**Figure 5.** Example of an image view

## 4.2 EDITING DATA

Active input fields are editable and are connected to the database. By clicking on the “Update” button the corresponding field is updated in the database table.

### EDITING CELL CONTOURS

To ensure accurate cutting, the user can manually revise the contours defined by the automatic segmentation methods. The contour of a cell can be drawn using the CAMIO interface. The new contour can be stored in the database by overwriting the originally generated one (**Figure 6**).

Figure 6. Cell view with cell contour.

## 4.3 EXPORTING DATA

The main functionality of CAMIO is to extract cells from the images of the high-content screening microscope. The software allows the user to export the contours of the cells of interest. Exported contours can be used for cutting cells by a microdissection microscope.

### EXPORTING CELL CONTOURS

For the above-defined purpose contours can be exported to a text file in ‘cell’ and ‘experiment’ views. In ‘cell’ view the contour of a single cell can be downloaded. To export a set of cells of a given phenotype each cell can be selected individually in ‘experiment’ view.

### (EXPORTING PDF)

This feature is not implemented yet.
